# Food-Derived High Arginine Peptides Promote Spermatogenesis Recovery in Busulfan Treated Mice

**DOI:** 10.3389/fcell.2021.791471

**Published:** 2021-12-21

**Authors:** Wenwen Liu, Lingfeng Zhang, Anning Gao, Muhammad Babar Khawar, Fengyi Gao, Wei Li

**Affiliations:** ^1^ College of Life Sciences, University of Science and Technology of China, Hefei, China; ^2^ Institute of Reproductive Health and Perinatology, Guangzhou Women and Children's Medical Center, Guangzhou Medical University, Guangzhou, China; ^3^ State Key Laboratory of Stem Cell and Reproductive Biology, Institute of Zoology, Chinese Academy of Sciences, Beijing, China; ^4^ College of Biology and Food, Shangqiu Normal University, Shangqiu, China; ^5^ Molecular Medicine and Cancer Therapeutics Lab, Department of Zoology, Faculty of Sciences, University of Central Punjab, Lahore, Pakistan

**Keywords:** food-derived peptides, Oyster peptides, Perilla purple peptides, spermatogenesis, oligoastheniaspermia

## Abstract

Food-derived peptides with high arginine content have important applications in medicine and food industries, but their potential application in the treatment of oligoasthenospermia remains elusive. Here, we report that high-arginine peptides, such as Oyster peptides and Perilla purple peptides were able to promote spermatogenesis recovery in busulfan-treated mice. We found that both Opp and Ppp could increase sperm concentration and motility after busulfan-induced testicular damage in mice. Further research revealed that Opp and Ppp might promote spermatogonia proliferation, which improved blood-testis barrier recovery between Sertoli cells. Taken together, these high-arginine peptides might be used as a medication or therapeutic component of a diet prescription to improve the fertility of some oligoasthenospermia patients.

## Introduction

Male infertility affects millions of people and, recently, has become an important human health issue (Boivin et al., 2007; Mascarenhas et al., 2012). Male infertility can be caused by various factors such as extreme heat exposure, oxidative stress, pesticides, radiation, immunosuppressive medicines, and other harmful chemicals (Bretveld et al., 2007; Bisht et al., 2017; Kesari et al., 2018; Perez-Garcia et al., 2020). The most common causes of male infertility are primary testicular dysfunction and oligoasthenospermia (Krausz, 2011). The implementation of appropriate infertility treatment is a necessity and priority globally (Ombelet et al., 2008; Schlegel et al., 2021). Food-derived peptides, peptides originating from plants and animals that are released during digestion or food processing, have been linked to a variety of health benefits (Erdmann et al., 2008). The therapeutic potential of food-derived peptides for the digestive, cardiovascular, immune, and nervous systems has been extensively investigated during the last few decades (Kitts and Weiler, 2003; Patil et al., 2015; Cicero et al., 2017; Chalamaiah et al., 2018). However, the effect of food-derived peptides on male fertility is still largely unknown.

Arginine (Arg), also known as L-arginine, plays important roles in ammonia detoxification, and nitric oxide and creatine production (Delage et al., 2010). Kim et al. found that the addition of 0.4% arginine to the diet of piglets from 7 to 21 days of age significantly upregulates their average daily weight gain by about 60% (Kim and Wu, 2004). Recent studies have shown that arginine is also involved in the regulation of reproductive processes in mammals. For instance, arginine has been shown to upregulate vascular endothelial growth factor 2 (VEGF2) transcriptional activity in the placenta of pregnant mice, thereby promoting maternal-fetal blood flow (Greene et al., 2012). The addition of arginine to the diet of gestating sows was found to significantly increase not only the number of surviving pups, but also the weight of the surviving pups after parturition (Mateo et al., 2007). In addition, Wu et al. found that the addition of arginine to boar rations did not change the ejaculate volume, but significantly increased the sperm concentration and viability (Wu et al., 2007). These results suggested that arginine might play an important role in mammalian reproduction and significantly improve the reproductive capacity of animals. However, human clinical interventions with L-arginine in its free amino acid form have resulted in endothelium-dependent vascular dilation in patients with endothelial dysfunction (Coman et al., 2008). When compared to free amino acids, high arginine peptides are more readily delivered into cells (Allolio et al., 2018). With low-molecular weight, these peptides are easily absorbed in the intestinal tract. Indeed, absorption of short-chain peptides is considered more effective than that of an equivalent amount of free amino acids and intact protein (Ji et al., 2017; Zamyatnin, 2018).

Oyster peptides (Opp) are oligopeptide-enriched hydrolysates obtained from oyster proteins (*Crassostrea gigas*), containing a high concentration of arginine (Supplementary Table S1) (Wang et al., 2010). A variety of functional active peptides have been found in oyster proteins, including antihypertensive, antifungal, and anticancer peptides (Defer et al., 2013; Gomez et al., 2019). Perilla purple peptides (Ppp) are obtained from an important crop in Asian countries: purple perilla (*Perilla frutescens L. Britt.*) seeds (Liu et al., 2020). The protein content, also rich in arginine, of purple perilla seeds can reach 23.7% (Longvah and Deosthale, 1998). Although it has been reported that Perilla purple seeds can be used to treat many diseases (Yu et al., 2017), the potential functions of Perilla purple seed peptides remain to be investigated.

In this study, we used a busulfan-induced non-obstructive azoospermic (NOA) mouse model to explore the possible effects of Opp and Ppp on spermatogenesis. We found that both Opp and Ppp could increase sperm concentration and motility by promoting the proliferation of spermatogonia, which in turn improves blood-testis barrier (BTB) recovery between Sertoli cells after busulfan treatment. Thus, it is expected that these high-arginine peptides might be used in the treatment of infertility in men who have received chemotherapy or been diagnosed with oligospermia or azoospermia.

## Materials and Methods

### Animals

Adult ICR male mice, purchased from SPF (Beijing) Biotechnology CO., Ltd, were maintained with The Animal Research Committee of the Institute of Zoology, Beijing, China. Mice were housed in 12 h alternating light/dark cycles, with free access to water and food. All experiments were conducted with the approval of the Animal Research Committee of the Institute of Zoology, Chinese Academy of Sciences. Mice were killed under standard protocols, and all efforts were made to minimize suffering.

### Design of Busulfan-Induced NOA Mouse Model and Treatment With Opp and Ppp

Mice were given a single round, intraperitoneal injection of either busulfan (35 mg/kg, Sigma, St. Louis, MO, United States) or DMSO (Sigma), according to previously described methods (Bucci and Meistrich, 1987; Qu et al., 2020). After 4 weeks, the testis weight, epididymal sperm count, and testis histology were evaluated and the phenotypes were checked. The remaining mice were then separated into three groups. The first group was given daily oral administration of 0.5 g/kg Oyster peptides, the second group was given daily oral administration of 0.5 g/kg Perilla purple peptides, and the control group was given daily oral administration of equal amounts of water. Opp and Ppp were provided by the Beijing Engineering Research Center of Protein and Functional Peptides. After 6 weeks, the testis weights, epididymal sperm count, and testis histology were evaluated.

### Sperm Analysis

After 10 weeks of busulfan treatment, the mice were sacrificed and epididymides were sampled for sperm analysis as previously described (Wang et al., 2014). In brief, the caudal epididymis was dissected from each group of mice. Spermatozoa were squeezed out from the caudal epididymis and incubated for 30 min at 37°C under 5% CO_2_. The incubated sperm medium was then diluted 1:500 and transferred to a hemocytometer for counting using a Primo Star microscope (Zeiss). The sperm motility assays were performed as previously described (Jimenez et al., 2010). Briefly, 10 μL aliquots of the sperm sample were taken before dilution and placed into 70 μm-deep glass cell chambers (Leja Products BV, NieuwVennep, Netherlands). The chambers were imaged using an Olympus BX51 microscope (Olympus, Tokyo, Japan) through a 20× phase objective and maintained at 37°C on a water bath. The viewing areas of each chamber were captured using a CCD camera. The samples were analyzed via computer-assisted semen analysis using the Mini-tube Sperm Vision Digital Semen Evaluation system. Different sperm motility parameters were analyzed.

### Immunofluorescence

Non-fixed sperm were spread on precoated slides with 10 μL for morphological observation and immunostaining. After air drying at room temperature, PBS (pH 7.4) was used to wash the slides three times, with 5 min of rest time between washes. The spermatozoa were then fixed in 4% (w/v) paraformaldehyde (PFA; P1110, Solarbio, Beijing, China) at room temperature for 10 min, and the slides were again washed with PBS three times, with 5 min of rest time between washes. After this, the slides were treated with 0.1% Triton X-100 for 10 min, rinsed with PBS three times, and blocked using 5% bovine serum albumin (BSA, AP0027, Amresco, Framingham, United States) in PBS for 30 min. The primary antibody was added to the slides and incubated at 4°C overnight. This was followed by incubation with FITC- or TRITC-conjugated secondary antibody at 1:200 for 1 h at 37°C. Next, the slides were washed in PBS, and the nuclei were stained with 4′,6-diamidino-2-phenylindole (DAPI; D3571, Life Technologies). Images were taken immediately using a LSM 780 microscope (Zeiss, Germany), LSM 880 Fast Ariyscan, or SP8 microscope (Leica, Germany). The antibodies used in this study are summarized in Table 1.

**TABLE 1:** Antibodies used for various experiments in this study.

Antibody	Source	Catalog number		Application
Goat anti-rabbit FITC	Zhong Shan Jin Qiao		ZF-0311	IF (1:200)
Goat anti-mouse FITC	Zhong Shan Jin Qiao		ZF-0312	IF (1:200)
Goat anti-mouse TRITC	Zhong Shan Jin Qiao		ZF-0313	IF (1:200)
Mouse anti-sp56	QED Bioscience		55101	IF (1:200)
Rabbit anti-WT1	Abcam		ab899901	IF(1:200);WB(1:1000)
Rabbit anti-SYCP3	Abcam		ab97672	IF(1:200);WB(1:1000)
Rabbit anti-DDX4	Abcam		ab27591	IF (1:400); WB(1:1000)
Rabbit anti-Ki67	Abcam		ab15580	IF (1:600)
Rabbit anti-ZO-1	Thermo Fisher Scientific		40-2200	IF (1:200)
Rabbit anti-β-catenin	Thermo Fisher Scientific		71-2700	IF (1:200)
Goat anti-PLZF	R&D system		AF2944	IF (1:200)
Mouse anti-GAPDH	ABclonal		AC002	WB (1:10000)

WB, western blotting; IF, immunofluorescence.

### Immunoblotting

Testes harvested from Opp, Ppp, and control mice were homogenized in cold RIPA buffer (Solarbio, R0010) and supplemented with 1 mM phenylmethylsulphonyl fluoride (PMSF, Amresco, 0754) and complete EDTA-free protease inhibitor cocktail (Roche Diagnostics, Rotkreuz, Switzerland, 04693116001). The homogenates were centrifuged at 12,000 rpm for 15 min. Protein lysates (25 µg) were separated by SDS-PAGE and transferred to a nitrocellulose membrane by electrolysis. Proteins obtained from lysates were separated by SDS-PAGE and transferred to polyvinylidene difluoride membranes (Millipore, Bedford, MA, United States). The membranes were then incubated in TBS-T (10 mM Tris-HCl, pH 7.4, 150 mM NaCl and 0.1% Tween-20) containing 5% non-fat milk at room temperature for 1 hour and stained with the appropriate primary and secondary antibodies, which are summarized in Table 1. After the final washes with TBS-T, the membranes were developed using the ODYSSEY Sa Infrared Imaging System (LI-COR Biosciences, Lincoln, NE).

### Tissue Collection and Histological Analysis

Testes from each group were dissected immediately after euthanasia, fixed in 4% paraformaldehyde (PFA; Solarbio, Beijing, China, P1110) for at least 24 h, and stored in 70% ethanol. They were then embedded in paraffin, cut into 5 μm sections, and mounted on glass slides. After deparaffinization, the slides were stained with hematoxylin and either eosin or periodic acid-Schiff (PAS) for histological analysis. The slides were scanned by Leica Aperio VESA8. The stages of seminiferous the epithelium cycle and spermatid development were detected as previously described (Hess and Renato de Franca, 2008).

### Statistical Analysis

All data are reported as the mean ± SD. GraphPadPrism9 software (GraphPad Software, La Jolla, CA, United States) was used for the graphing and statistical analysis of data to determine whether the data conform to the normal distribution. The statistical significance of the differences between the various groups was measured by Student’s *t*-test with an unpaired, two-tailed distribution. The statistical significances were defined as: *, *p* < 0.05; **, *p* < 0.01; ***, *p* < 0.001; ****, *p* < 0.0001.

## Results

### Establishment of the Busulfan-Treated NOA Mouse Model

**FIGURE 1:**
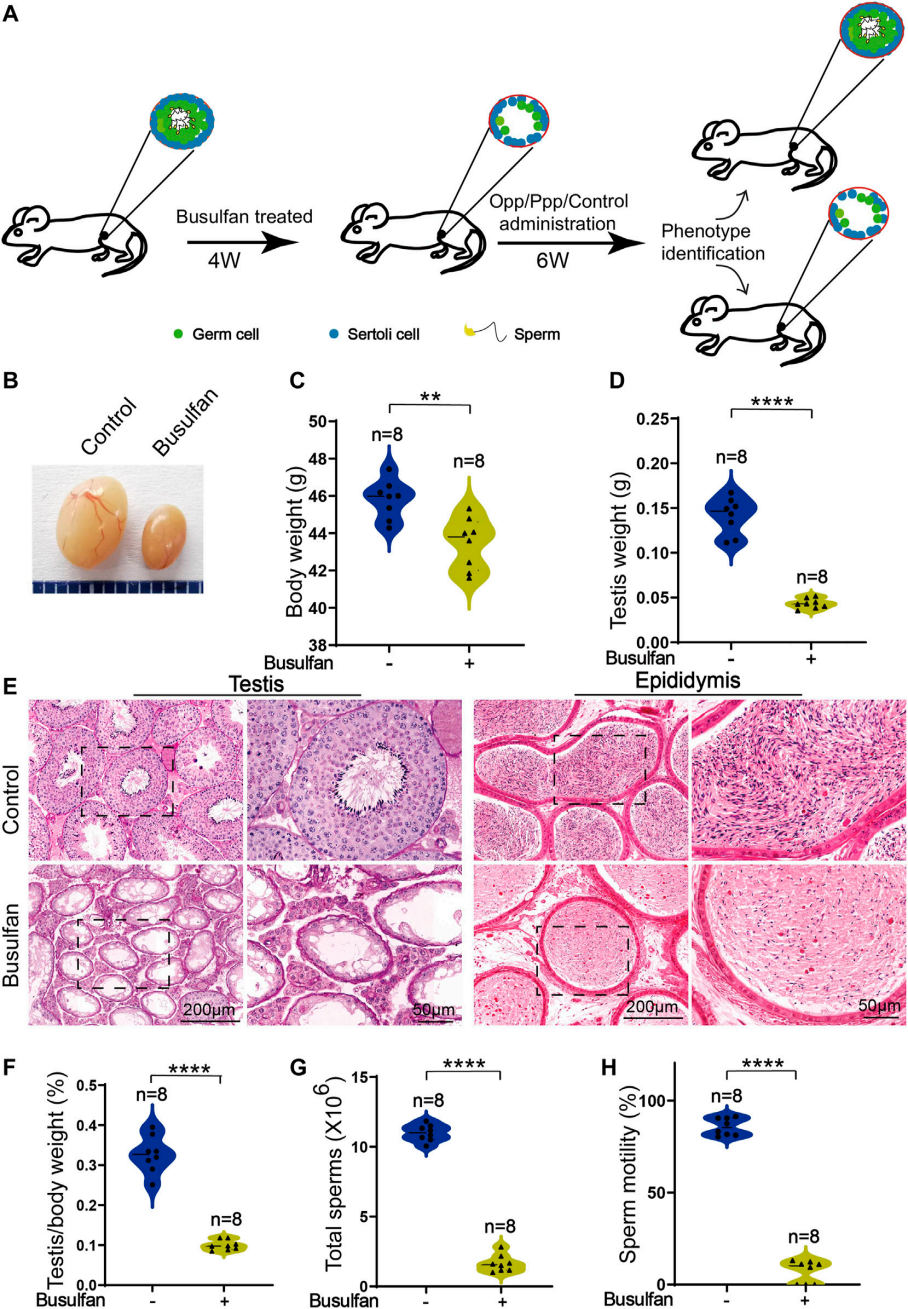
Busulfan-induced germ cell loss in mouse testes. **(A)** Schematic depiction of the experimental procedure used for high arginine peptide administration. **(B)** Picture of the testes of DMSO- and busulfan-treated mice, where the busulfan-treated testes were smaller than those of the DMSO-treated testes. The body **(C)** and testis **(D)** weights of DMSO- and busulfan-treated mice. **(E)** Histology of the seminiferous tubules and the cauda epididymis of DMSO- and busulfan-treated male mice. **(F)** The ratio of testis weight/body weight in DMSO- and busulfan-treated male mice. **(G)** Sperm counts **(G)** and motility **(H)** in the caudal epididymis of busulfan- and DMSO-treated male mice. Data are presented as the mean ± SD. The statistical significance of the differences between the mean values for the different groups was measured by Student’s *t*-test with unpaired, two-tailed distribution. **p < 0.01, ****p < 0.0001.

To explore the effects of food-derived high arginine peptides on the spermatogenesis process, an NOA mouse model was developed with treatment by the genotoxic agent, busulfan (Bucci and Meistrich, 1987). Briefly, the mouse model was established by single intraperitoneal administration of busulfan in wild-type 8-week-old male mice, or DMSO as control group. After 4 weeks of administration, the success rate of modeling was then detected. Subsequently, busulfan-induced NOA mouse were treated with high arginine peptides for six consecutive weeks, and the testis histology was then detected (Figure 1A).

After 4 weeks of busulfan treatment, the body weights of the busulfan-treated mice were considerably lower than that of the control group (Figure 1C). To learn more about the phenotypes of busulfan-treated mice, we then examined the testis structure from both macroscopic and histological standpoints. Here, we found significant differences in the testis size, weight, and testis/body weight ratio between the control and busulfan-treated mice (Figures 1B,D,F). Hematoxylin and eosin (H&E) staining revealed that the seminiferous tubules of the busulfan-treated mice were almost empty. The spermatozoa in the cauda epididymis were analyzed, and we found that busulfan-treated mice had no or few spermatozoa in the epididymal lumen (Figure 1E). We also examined the spermatozoa released from the cauda epididymis of the busulfan-treated mice and found that the sperm count and motility were much lower than that of the control mice (Figures 1G,H). Thus, we successfully established a busulfan-induced azoospermic mice model.

### Opp and Ppp Promote Spermatogenesis Recovery in Busulfan-Induced Mice

**FIGURE 2:**
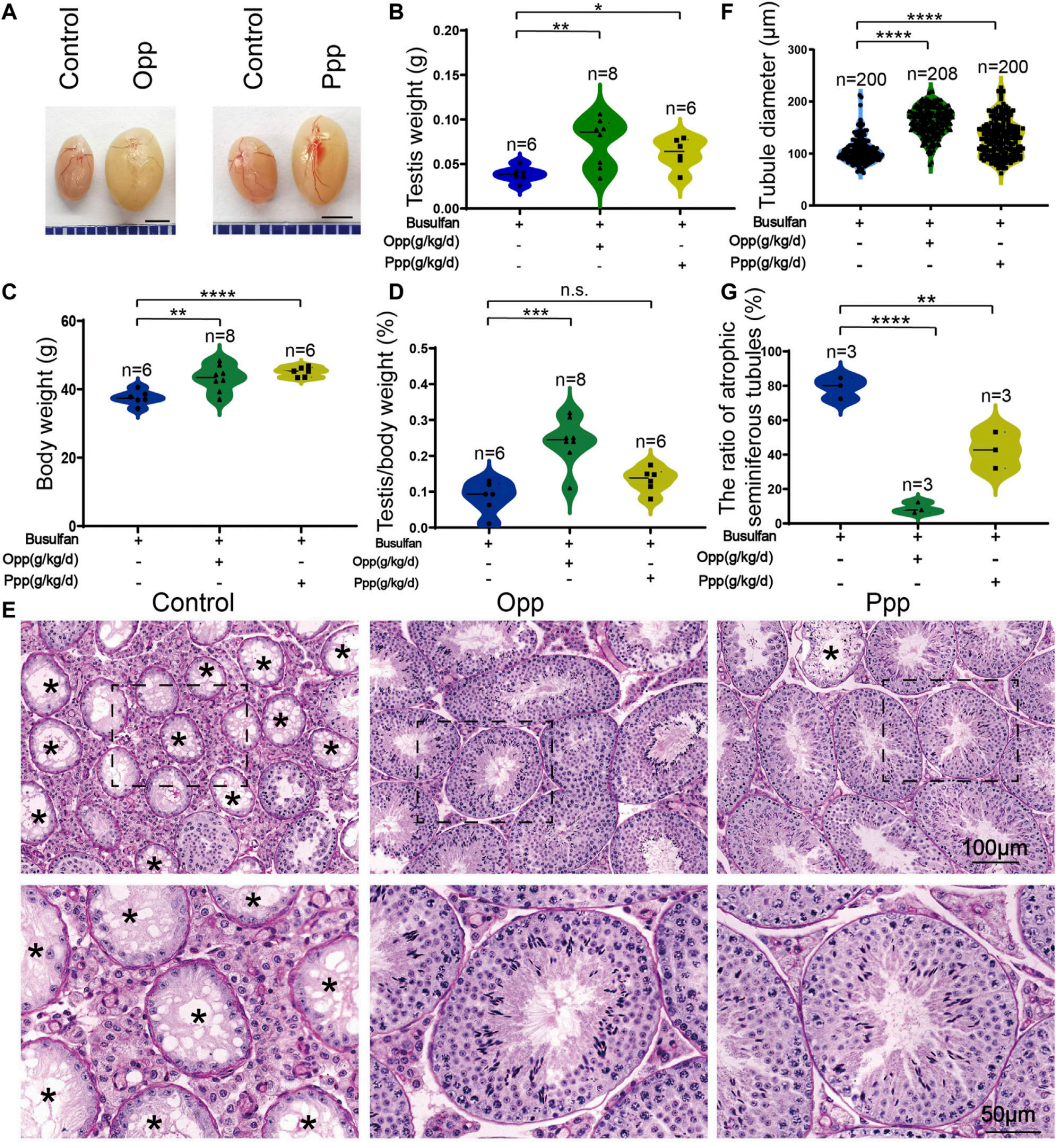
Opp and Ppp promotes spermatogenesis recovery after busulfan treatment in mice. **(A)** The effects of Opp and Ppp on busulfan treatment mice after 6 weeks of intragastric administration of treatment in control testis (vehicle-treated, *n* = 6), Opp-treated testis (Opp-treated, *n* = 8), and Ppp-treated testis (Ppp-treated, n = 6). The testis**(B)** and body **(C)** weight of control, Opp-, and Ppp-treated mice. **(D)** The ratio of testis weight/body weight in control, Opp-, and Ppp-treated male mice. **(E)** The histology of the seminiferous tubules of control, Opp- and Ppp-treated male mice. Asterisk indicates the atrophic tubules. **(F)** The diameter of the seminiferous tubules in the control, Opp-, and Ppp-treated male mice. **(G)** The ratio of atrophic seminiferous tubules in control, Opp-, and Ppp-treated. Data are presented as mean ± SD. The statistical significance of the difference between the mean values for each group was measured by Student’s *t*-test with unpaired, two-tailed distribution. *p < 0.05, **p < 0.01, ***p < 0.001, and ****p < 0.0001.

To characterize the potential functions of Opp and Ppp on spermatogenesis, the busulfan-treated mice were further treated with Opp and Ppp for another 6 weeks, while the control group was only administrated with water (vehicle of peptide administration) (Figure 1A). We found that the testes size (Figure 2A) and weight (Figure 2B) of Opp- and Ppp-treated mice were significantly increased compared with the control mice. Interestingly, the body weight of both the Opp and Ppp groups were also significantly increased (Figure 2C). Thus, there were no significant differences in the testis/body weight ratio between the control and the Ppp-treated groups (Figure 2D).

**FIGURE 3:**
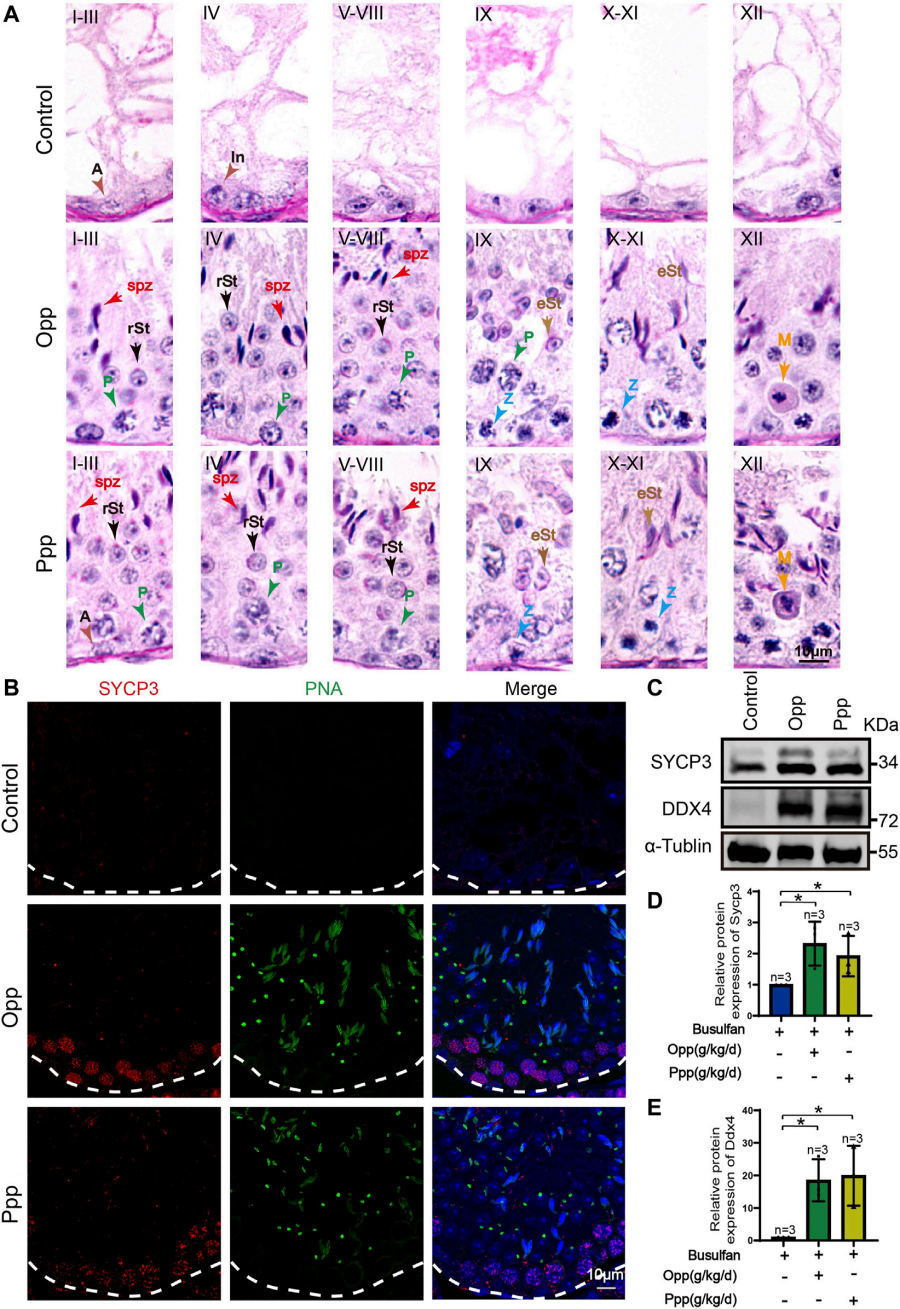
Spermatogenesis recovery after Opp and Ppp administration. **(A)** PAS staining of testes sections of control, Opp- and Ppp-treated mice. A: type A spermatogonia, In: intermediate spermatogonia, P: pachytene spermatocyte, Z: zygotene spermatocyte, M: meiotic spermatocyte, rSt: round spermatid, eSt: elongating spermatid, and spz: spermatozoa. **(B)** Representative immunofluorescence images showing newborn germ cells stained with SYCP3 (red) and PNA (green). **(C)** Immunoblotting of SYCP3 and DDX4 was performed in the control, Opp-, and Ppp-treated male mice testis. **(D)** Quantification of the relative protein levels of SYCP3 in Opp- and Ppp-treated groups using the Odyssey software. **(E)** Quantification of the relative protein levels of DDX4 in Opp- and Ppp-treated groups using the Odyssey software. Data are presented as the mean ± SD. The statistical significance of the differences between the mean values for each group was measured by Student’s *t*-test with unpaired, two-tailed distribution. *p < 0.05.

The H&E histological analysis further revealed that thinner seminiferous epithelia were found in the testes of control mice while most of the tubules in Opp- and Ppp-treated testes contained all stages of spermatid development in the seminiferous epithelium (Figure 2E). The results of the histological analysis showed that the diameters of round seminiferous tubules in the Opp- and Ppp-treated groups were observably thickened compared to the control group (Figure 2F). In the control group, 80% of the atrophic seminiferous tubules remained, while the number of atrophic seminiferous tubules decreased by less than 20% in the Opp-treated groups and 40% in the Ppp-treated groups (Figure 2G). Further staining with periodic acid-schiff (PAS)-hematoxylin demonstrated that all stages of germinal epithelium from spermatogonia to spermatozoa were recovered in Opp- and Ppp-administered groups compared to the almost empty control groups (Figure 3A). Close examination by peanut agglutinin (PNA) staining (to show the development of acrosome) found that acrosomes were well-developed in the Opp- and Ppp-treated groups, but not in the control mice (Figure 3B). Consistent with the PNA staining results, the expression of SYCP3, a meiotic cell-specific protein, was also dramatically increased in the seminiferous tubules of the Opp- and Ppp-treated groups compared to those of the control group (Figures 3C,D). These results were further supported by western-blot detection of DDX4 (Figures 3C,E). Taken together, these results demonstrate that both Opp and Ppp can promote the recovery of impaired meiosis and spermatogenesis after busulfan treatment.

**FIGURE 4:**
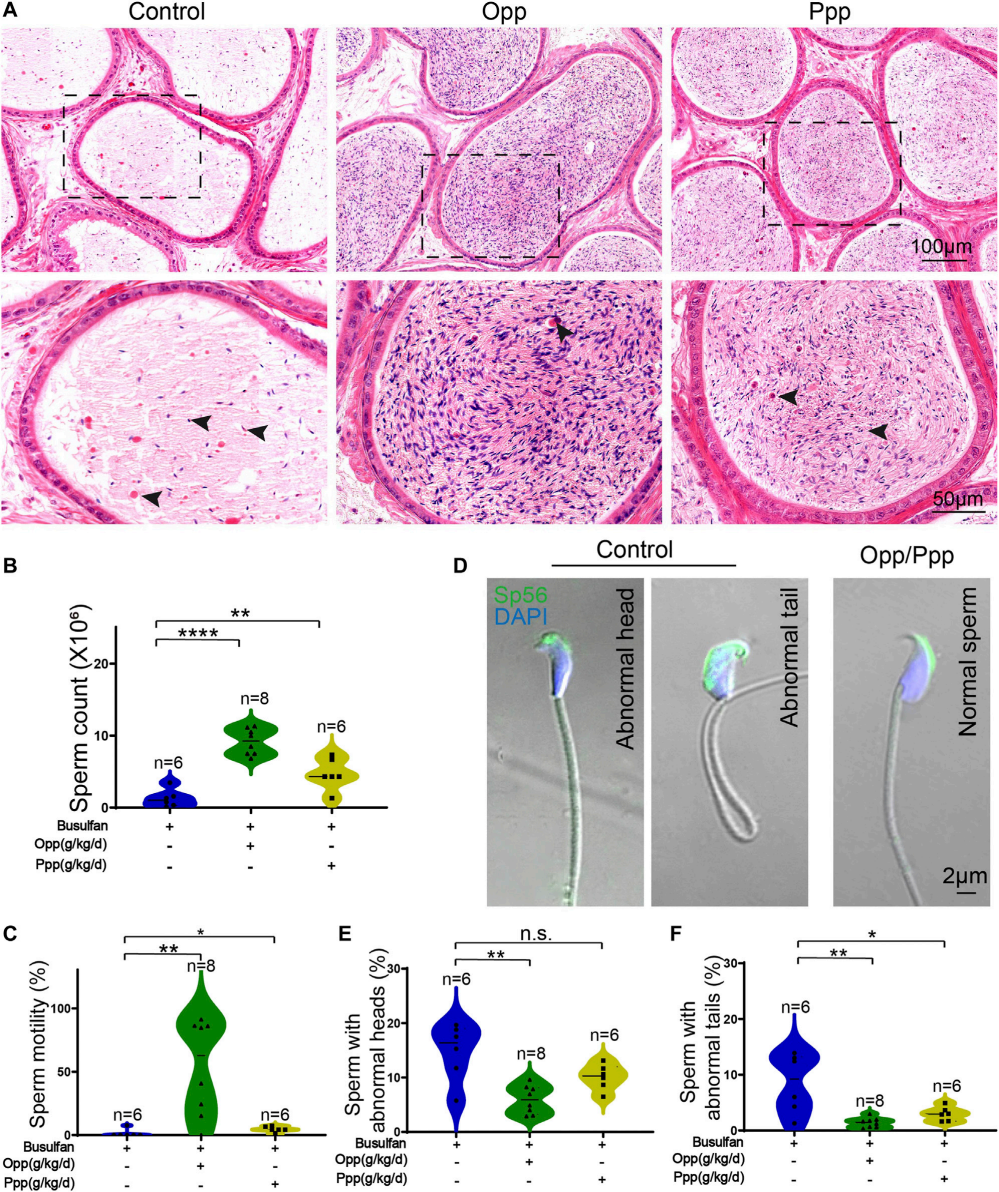
Opp and Ppp treatment increase spermatozoa counts and motilities after busulfan treatment in mice. **(A)** H&E staining of the caudal epididymis from control, Opp-, and Ppp-treated mice. Black arrow heads indicate abnormal spermatozoa. **(B)** The total number of sperm from the cauda epididymis was significantly increased in Opp-treated and Ppp-treated mice. **(C)** Motile sperm in the control, Opp-, and Ppp-treated mice. **(D)** Confocal images to show Sp56 staining of the control, Opp-, and Ppp-treated spermatozoa with abnormal head or coiled flagella. **(E)** Quantification of spermatozoa with abnormal heads in the control, Opp-, and Ppp-treated mice. **(F)** Quantification of spermatozoa with abnormal tails in the control, Opp-, and Ppp-treated mice. Data are presented as the mean ± SD. The statistical significance of the differences between the mean values for the different groups was measured by Student’s t-test with unpaired, two-tailed distribution. *p < 0.05, **p < 0.01.

It has previously been reported that busulfan treatment leads to abnormalities in mice sperm heads (Bucci and Meistrich, 1987). To determine the effect of the Opp and Ppp treatment on these abnormalities, we used H&E staining to examine transverse sections of the cauda epididymis. Here, we discovered that the Opp- and Ppp-treated animals had more epididymal sperm than the control mice, which had only a few aberrant spermatozoa and numerous detached premature germ cells in the epididymal lumen (Figure 4A). We further examined the spermatozoa released from the caudal epididymis and found the sperm count in Opp- and Ppp-treated mice was dramatically increased compared with that of the control mice (Figure 4B). In addition, sperm motility was measured by computer-assisted semen analysis (CASA). We found that the Opp-treatment could promote sperm motility significantly when compared with that of the control mice (Figure 4C). Single-sperm immunofluorescence was utilized to examine the morphological properties of the spermatozoa using the acrosome-specific marker, Sp56 (Zp3r). Morphological evaluation revealed that many spermatozoa from control mice had more irregularly-shaped sperm heads and tails compared to the Opp-treatment and Ppp-treatment groups (Figures 4D–F). These results suggest that the administration of both Opp and Ppp could at least partially restore epididymal sperm morphology, spermatozoa count, and motility.

### Opp and Ppp Treatment May Promote the Proliferation of Spermatogonia

**FIGURE 5:**
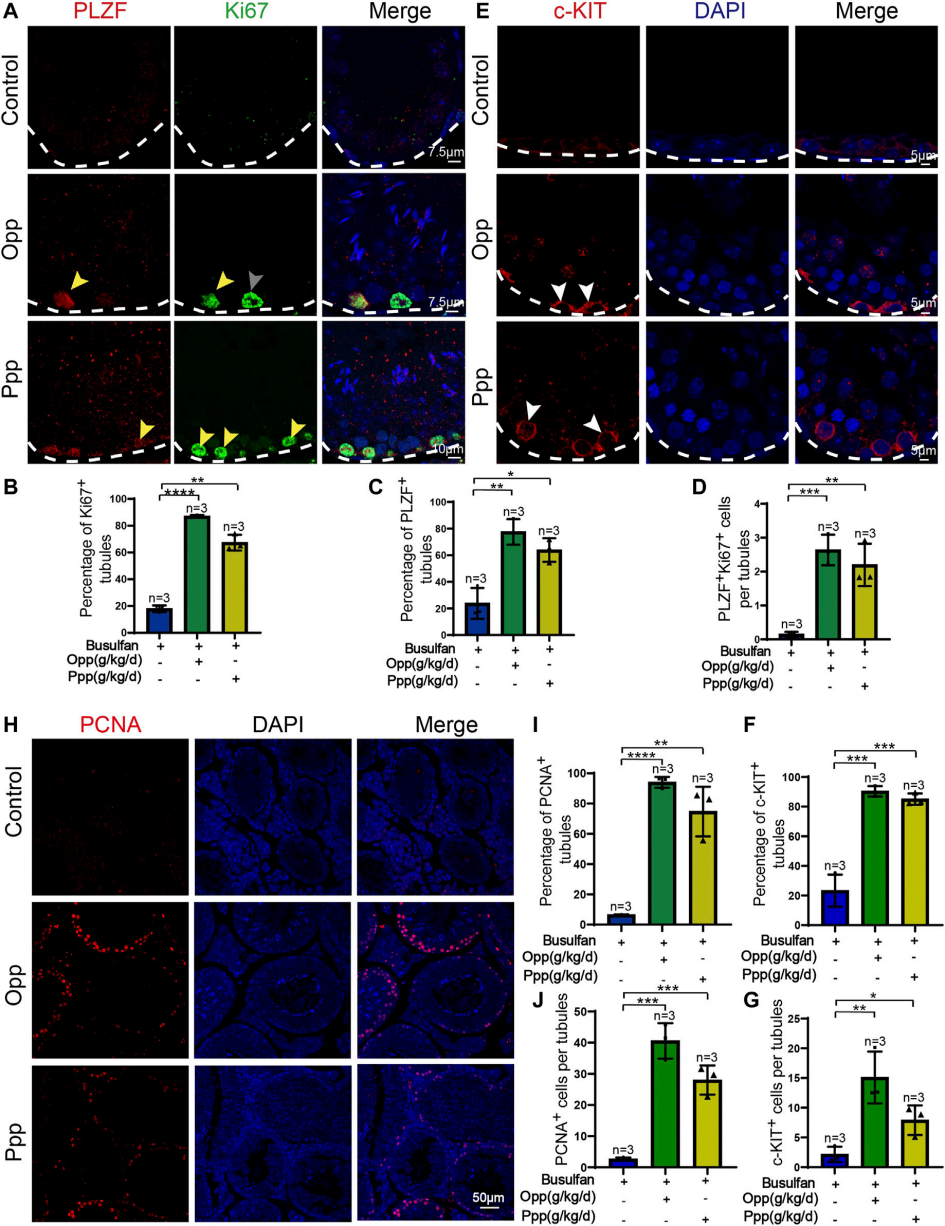
Opp and Ppp promote spermatogonia proliferation after busulfan treatment in mice. **(A)** Co-immunostaining of PLZF and Ki67 in the seminiferous tubule of control, Opp-, and Ppp-treated testes. White arrow signifies seminiferous tubules containing only PLZF-positive spermatogonia. Gray arrow signifies seminiferous tubules containing only Ki67-positive spermatogonia. Yellow arrow signifies seminiferous tubules containing both PLZF and Ki67 positive spermatogonia. **(B)** The percentage of Ki67-positive tubules in control, Opp-, and Ppp-treated testes. **(C)** The percentage of PLZF-positive tubules of control, Opp-, and Ppp-treated testes. **(D)** Quantification of Ki67-and PLZF-double positive cells per tubule. **(E)** Immunofluorescence staining of c-KIT (red) and DAPI (blue) in the seminiferous tubule of control, Opp-, and Ppp-treated testes. **(F)** The percentage of c-KIT-positive tubules in control, Opp-, and Ppp-treated testes. **(G)** Quantification of c-KIT positive cells per tubules of control, Opp-, and Ppp-treated testes. **(H)** Representative immunofluorescence images using the PCNA antibody of the seminiferous tubules of control, Opp-, and Ppp-treated testes. **(I)** The percentage of PCNA-positive tubules in control, Opp-, and Ppp-treated testes. **(J) **Quantification of PCNA positive cells per tubule in control, Opp-, and Ppp-treated testis. Data are presented as mean ± SD. The statistical significance of the differences between the mean values for each group was measured by Student’s *t*-test with unpaired, two-tailed distribution. *p < 0.05, **p < 0.01, ***p < 0.001.

Spermatogenesis starts from spermatogonia, which are the only stem cells in the germline that undergo either self-renewal or differentiation (Kanatsu-Shinohara and Shinohara, 2013). It has been reported that treatment with busulfan kills most of the germ cells, but not spermatogonia (Bucci and Meistrich, 1987). Thus, we further investigated whether the therapeutic effects of Opp and Ppp were related to spermatogenesis from the endogenous population of spermatogonia cells. Immunofluorescence staining was performed to examine the expression of the undifferentiated spermatogonia marker, promyelocytic leukaemia zinc finger (PLZF), and proliferating cell nuclear antigen (Ki67), a marker of cell proliferation (Wang et al., 2019). We found that the number of PLZF^+^Ki67^+^ spermatogonia was dramatically increased in Opp and Ppp-treated seminiferous tubules (Figures 5A–D), while almost no PLZF^+^Ki67^+^ spermatogonia were detected in the control mice (Figures 5A–D).

Furthermore, we examined differentiating spermatogonia markers, such as c-KIT, in every mouse group (Figure 5E). In each testicular cross-section of the Opp- and Ppp-treated groups, the number of c-KIT positive cells was higher than that of the control mice (Figures 5F,G). To further confirm the effects of Opp and Ppp on germ cell proliferation, we examined another general cell proliferation marker, proliferating cell nuclear antigen (PCNA). Here, we found that PCNA positive cells were rarely detected in the control seminiferous tubules, while the PCNA positive cells were increased in Opp- or Ppp-treated tubules (Figures 5H–J). Thus, all these results suggest that Opp and Ppp might promote spermatogonia proliferation, and restore spermatogenesis.

### Opp and Ppp Treatments Promote the Recovery of BTB Integrity

**FIGURE 6:**
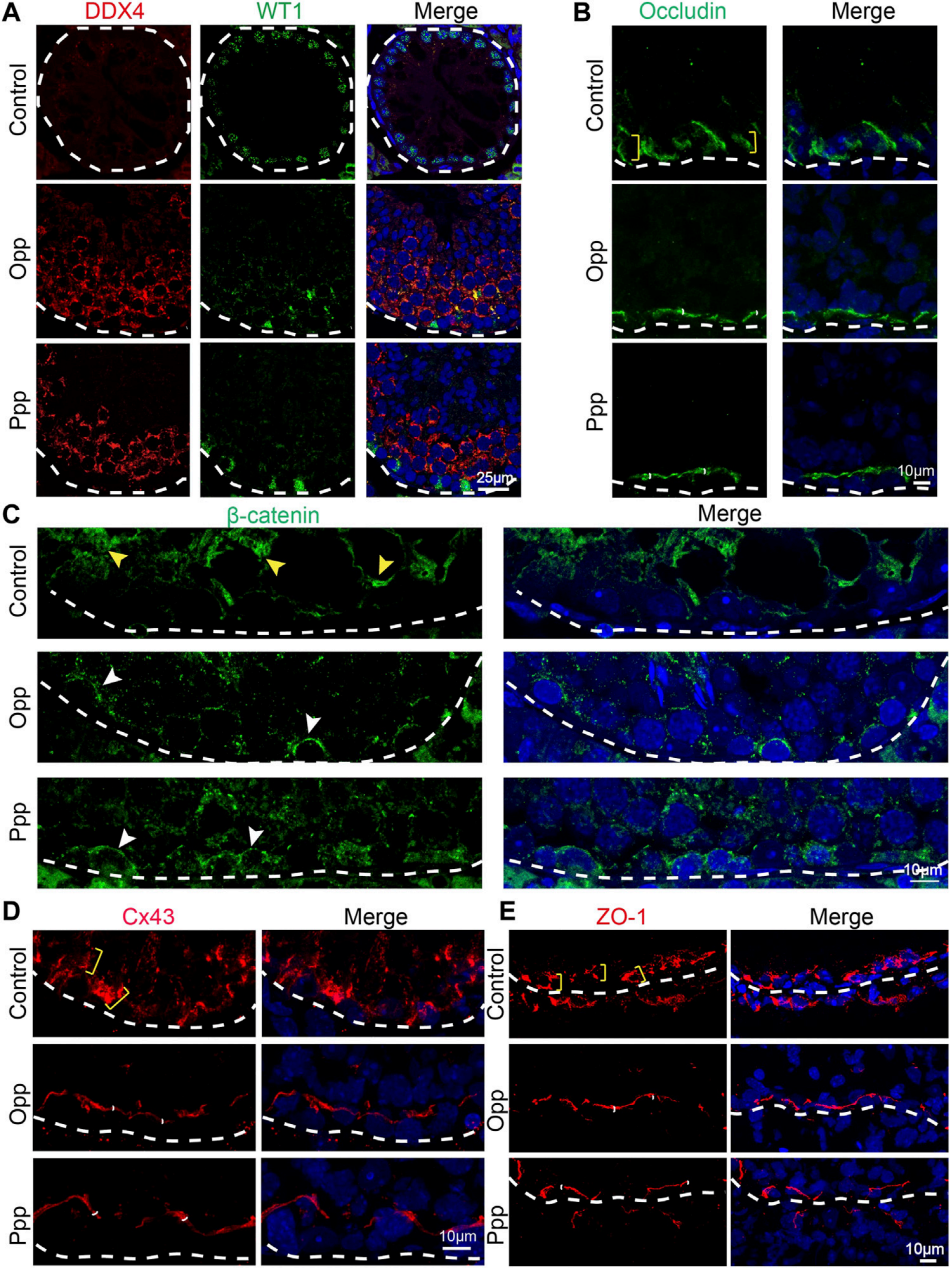
Opp and Ppp promote BTB recovery after busulfan treatment in Sertoli cells. **(A)** Immunofluorescence staining of control, Opp-, and Ppp-treated testes with anti-WT1 antibody (Sertoli cell, green), anti-DDX4 antibody (germ cell, red) and DAPI (blue). White dashed line depicts the basement membrane. **(B)** Immunofluorescence staining of control, Opp-, and Ppp-treated testes with anti-occludin antibody (BTB, green) and DAPI (blue). Yellow scale markers signify diffusively-distributed occludin signals. White scale markers depict tightly-distributed occludin signals around Sertoli and germ cells. **(C)** Immunofluorescence staining of control, Opp-, and Ppp-treated testes with anti-β-catenin antibody (BTB, green) and DAPI (blue). Yellow arrow heads signify diffusively-distributed β-catenin signals; white arrow heads signify tightly-distributed β-catenin signals around Sertoli and germ cells. **(D)** Immunofluorescence staining of control, Opp-, and Ppp-treated testes with anti-Cx43 antibody (BTB, green) and DAPI (blue). Yellow scale markers depict diffusively distributed Cx43 signals; white scale markers depict tightly-distributed Cx43 signals around Sertoli and germ cells. **(E)** Immunofluorescence staining of control, Opp-, and Ppp-treated testes with anti-ZO-1 antibody (BTB, red) and DAPI (blue). Yellow arrow heads label diffusively-distributed ZO-1 signals. White arrow heads label tightly-distributed ZO-1 signals.

It has been reported that busulfan treatment might also affect the function of Sertoli cells (Cai et al., 2016; Panahi et al., 2020). Therefore, we then examined the impact of Opp and Ppp on Sertoli cells by detecting WT1, a Sertoli cell-specific marker (Rotgers et al., 2018). After busulfan treatment, DDX4, a germ cells marker, was barely detected in the seminiferous tubules, while only WT1 positive cells were found at the basement layer of the tubules in the control group (Figure 6A). In contrast, after treatment with either Opp or Ppp, a vast number of DDX4 positive cells were found, suggesting the germ cells have been at least partially restored (Figure 6A). The essential function of Sertoli cells is to form the BTB, which not only separates seminiferous tubules into the basal and luminal compartments, but also provides a specialized microenvironment for spermatogenesis (Cheng and Mruk, 2012).

To explore the effects of Opp and Ppp on the BTB after busulfan treatment, the expression and localization of some BTB component proteins such as occludin, basal ectoplasmic specialization β-catenin, connexin 43 (Cx43) and junction proteins Zona occludin 1 (ZO-1) were examined by immunofluorescent analysis. In the control group testes, occludin, β-catenin, Cx43 and ZO-1 were all still diffusively-distributed (Figures 6B–E), while in both Opp- and Ppp-treated testes, ZO-1, β-catenin, occludin, and Cx43 were all localized tightly to the BTB sites, which were adjacent to the base of the membrane (Figures 6B–E). Together, these results suggest that Opp and Ppp might either directly or indirectly promote BTB recovery after busulfan treatment.

## Discussion

**FIGURE 7:**
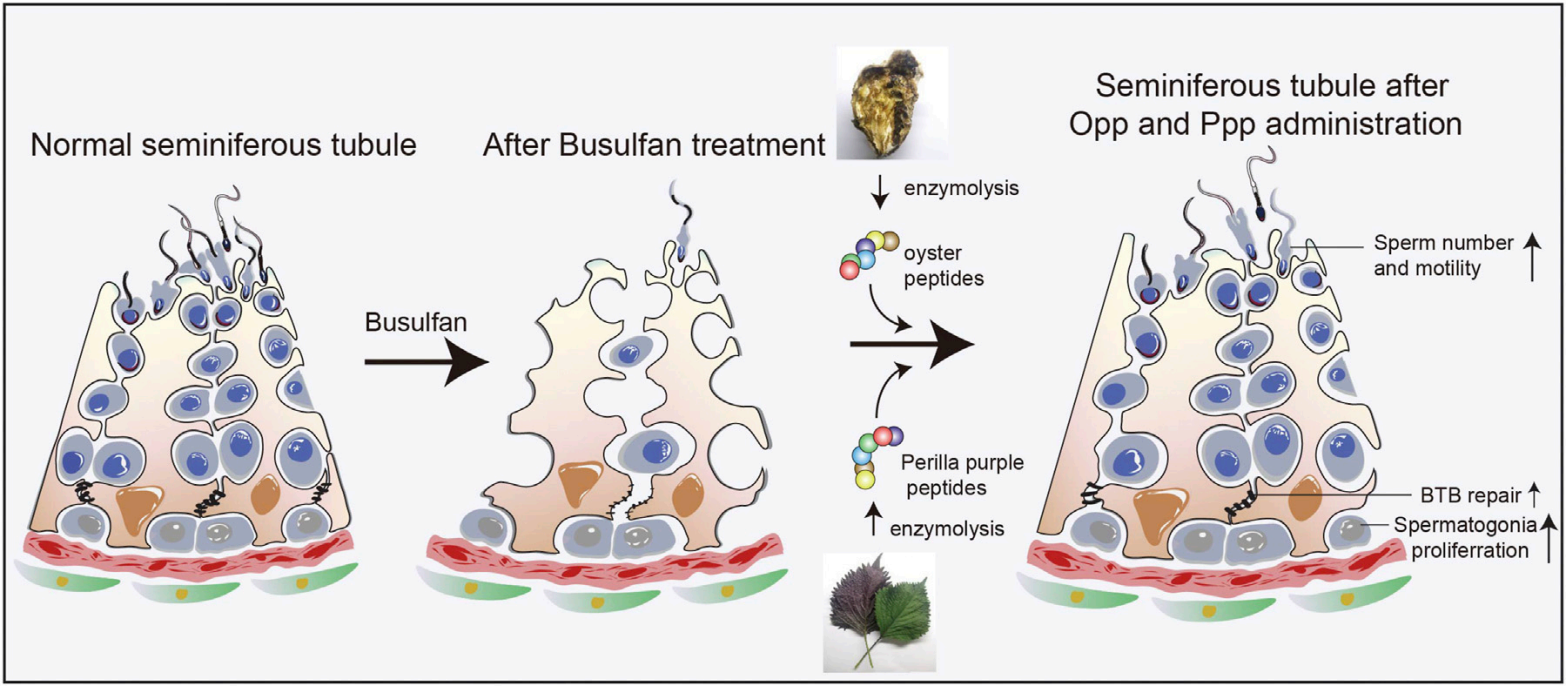
Proposed model for the functional role of Opp and Ppp on spermatogenesis. Food-derived high arginine peptides may promote spermatogonia proliferation, promoting BTB recovery between Sertoli cells, and thus increasing sperm concentration and motility after busulfan treatment.

Mammalian spermatogenesis is a multi-step process that is highly sensitive to chemotherapy and radiotherapy, as most patients lose their fertility permanently or transiently after chemotherapy or radiotherapy. It can take more than 5 years to regain the ability to impregnate, even if lost transiently (Brydøy et al., 2010). Despite the use of numerous treatments, such as aided antioxidants and hormonal therapy, to improve sperm quality and raise the odds of conception, curing the malfunction of the spermatogenic microenvironment remains a difficult task (Meistrich et al., 1999; Ebisch et al., 2007; Ombelet et al., 2008). Here, we found that the food-derived high arginine peptides, Opp and Ppp, could restore spermatogenesis by activating endogenous spermatogonia proliferation after busulfan treatment. (Figure 7) Moreover, sperm count and motility were at least partially restored, suggesting these peptides, especially Opp, may be a good choice for the recovery of patients who have undergone chemotherapy and radiotherapy.

Busulfan is a myeloablative alkylating drug that has been frequently utilized to create a non-obstructive azoospermia (NOA) mouse model and is commonly associated with the development of protracted or permanent infertility (Costabile, 1993; Levine et al., 2010). Mouse fertility was lost within 4 weeks of starting busulfan treatment, and their fertility could be restored after at least 30 weeks (Bucci and Meistrich, 1987; Zohni et al., 2012). The mechanism of busulfan-induced sterility is primarily through the alkylation of DNA, disruption of vimentin filament distribution, impeding spermatogonia stem cell proliferation and differentiation, and promoting spermatogonia stem cell dormancy (Zhao Y et al., 2020; Chen et al., 2021). Busulfan treatment also leads to oligoasthenospermia, which is characterized by sperm head abnormalities and reduced sperm motility (Bucci and Meistrich, 1987; Zhao.X et al., 2020). We found that both sperm number and motility were significantly increased after Opp and Ppp treatment, especially in the Opp supplement groups, compared to that of the control group (Figure 3C). Interestingly, the body weights were also recovered after Opp and Ppp were administered compared to the control mice (Figure 2C). Geiger et al. demonstrated that intracellular L-arginine concentrations directly impact metabolic fitness (Geiger et al., 2016). Aside from the high arginine content, both Opp and Ppp contained large amounts of proteinogenic amino acids (particularly glycine, proline, glutamate, leucine, and lysine), which are essential for DNA and protein syntheses in cells or energy metabolism in tissues (Ma et al., 2017). Therefore, the body weights were increased compared to the control mice. It is worth noting that Opp and Ppp treatment might promote BTB recovery after busulfan treatment. Since the Sertoli cells and germ cells form a microenvironment to support spermatogonia stem cell development, the meiosis of spermatocytes, and spermatogenesis (Wen et al., 2018; Wu et al., 2020), all these results suggest that these two types of peptides might promote the regeneration of the seminiferous epithelium, which had been impaired by busulfan treatment.

Food-derived bioactive protein peptides or hydrolysates have been widely investigated by many researchers due to their abundance in biological activities. Multiple studies have demonstrated that protein hydrolysates or peptides can be obtained from a wide range of food protein sources, such as milk, egg, fish, crabs, shrimp, sea cucumbers, oysters, mussels, chlorella (algae), spirulina, rice, soybeans, corn, common beans, chickpeas, and grape seed (Kim et al., 2000; Kannan et al., 2011; Beaulieu et al., 2013). In addition, they have been reported to possess a wide range of bioactivities, including immunomodulatory, anticancer, antihypertensive, antioxidant, anti-inflammatory, mineral-binding, opiate, antilipemic, osteoprotective, and antimicrobial effects (Bhat et al., 2015). In addition to bioactivities, food-derived protein hydrolysates (peptides) possess various physicochemical properties, including solubility, lipid binding, foaming, and emulsification properties. However, the potential application of food-derived bioactive peptides on oligoasthenospermia treatment has been severely understudied.

Opp and Ppp are both food-derived peptides that have a high content of arginine. Peptides from oysters have been reported to enhance spleen lymphocyte and macrophage proliferation (Cai et al., 2013). Oral administration of oyster meat hydrolysates by alcalase could induce aphrodisiac activities and consequently enhance the sexual behavior in normal male mice (Zhang et al., 2019). According to Jin et al., Opp can help the aged male with cyclophosphamide-induced partial androgen insufficiency by increasing testosterone production (Jin et al., 2021). Although hydrolyzed peptides from purple perilla (*Perilla frutescens L. Britt.*) seeds have been reported to improve muscle synthesis and exercise performance in mice, few studies have tried to address its potential effect on the reproductive system (Liu et al., 2020). Even though both Opp and Ppp have high levels of arginine, they differ in the amount of arginine and, therefore, have different phenotypes. The arginine content of Opp is 4.856%, while Ppp can be as high as 9.81% (Supplementary Table S1). The efficiency of Opp is higher than that of Ppp (Figures 2, 3, 6), suggesting that the concentration of arginine may not be the unique reason for spermatogenesis restoration after busulfan treatment. In this way, some active peptides might exist in Opp, which still need to be further identified. Our studies provide a novel strategy to discover new food-derived bioactive peptides from vast animal and plant-based resources and open a promising way to treat some male infertilities.

## Data Availability

The original contributions presented in the study are included in the article/Supplementary Material, further inquiries can be directed to the corresponding authors.
