# Supplementary material for: Food-Derived High Arginine Peptides Promote Spermatogenesis Recovery in Busulfan Treated Mice
Source: Front Cell Dev Biol. 2021 Dec 21;9:791471. doi: 10.3389/fcell.2021.791471 (PMC8724571; doi:10.3389/fcell.2021.791471)
Supplement: Supplementary file 1 [file DataSheet1.PDF]

Table S1.

The amino acid composition of Oyster peptides and Perilla purple peptides

Amino acid	Opp (g/100g)	Ppp (g/100g)
Asp	6.054	7.88
Thr	2.491	3.03
Ser	3.138	3.99
Glu	9.761	14.84
Gly	4.66	3.88
Ala	4.399	3.87
Val	2.592	3.65
(Cys)2	0.28	1.70
Met	1.466	2.17
Ile	2.638	2.86
Leu	4.76	5.31
Tyr	1.728	3.09
Phe	1.373	4.18
His	1.362	2.32
Lys	4.626	3.12
Arg	4.856	9.81
Pro	2.377	3.30
Total	58.561	78.97

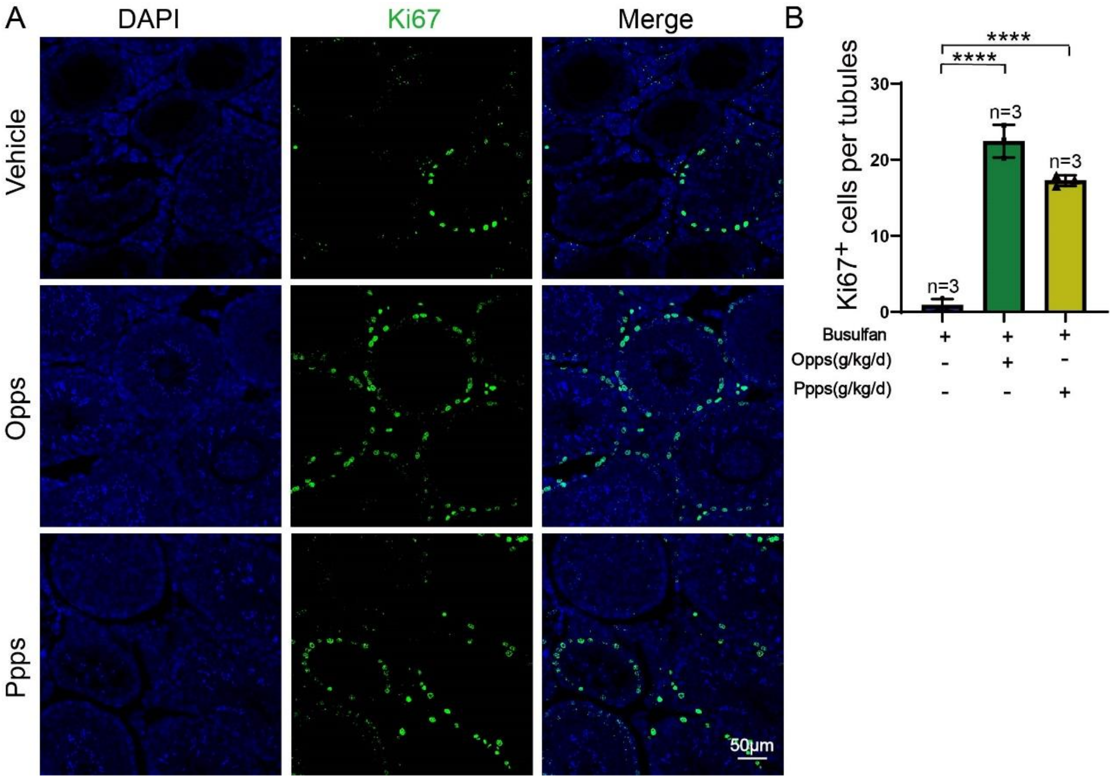

Figure S1. Opps and Ppps promote spermatogonia proliferation after busulfan treatment in mice. (**A**) Immunofluorescence staining of Opp-treated, Opp-treated and the control testis with ki67. (**B**) Quantification of ki67 positive cell numbers per seminiferous tubule. Data are presented as mean ± SD. The statistical significance of the differences between the mean values for the different groups was measured by Student's t-test with unpaired, two-tailed distribution.
